# Gaming New Zealand’s Emergency Department Target: How and Why Did It Vary Over Time and Between Organisations?

**DOI:** 10.15171/ijhpm.2019.98

**Published:** 2019-11-03

**Authors:** Tim Tenbensel, Peter Jones, Linda Maree Chalmers, Shanthi Ameratunga, Peter Carswell

**Affiliations:** ^1^Faculty of Medical and Health Sciences, The University of Auckland, Auckland, New Zealand.; ^2^Auckland District Health Board Faculty of Medical and Health Sciences, University of Auckland, Auckland, New Zealand.; ^3^Auckland District Health Board, Auckland, New Zealand.; ^4^School of Population Health, Faculty of Medical and Health Sciences, University of Auckland, Auckland, New Zealand.

**Keywords:** Gaming, Targets, Performance Management, Emergency Departments, New Zealand

## Abstract

**Background:** Gaming is a potentially dysfunctional consequence of performance measurement and management systems in the health sector and more generally. In 2009, the New Zealand government initiated a Shorter Stays in Emergency Department (SSED) target in which 95% of patients would be admitted, discharged or transferred from an emergency department (ED) within 6 hours. The implementation of similar targets in England led to well-documented practices of gaming. Our research into ED target implementation sought to answer how and why gaming varies over time and between organisations.

**Methods:** We developed a mixed-methods approach. Four organisation case study sites were selected. ED lengths of stay (ED LOS) were collected over a 6-year period (2007-2012) from all sites and indicators of target gaming were developed. Two rounds of surveys with managers and clinicians were conducted. Interviews (n=68) were conducted with clinicians and managers in EDs and the wider hospital in two phases across all sites. The interview data was used to develop explanations of the patterns of variation across time and across sites detected in the ED LOS data.

**Results:** Our research established that gaming behaviour – in the form of ‘clock-stopping’ and decanting patients to short-stay units (SSUs) or observation beds to avoid target breaches – was common across all 4 case study sites. The *opportunity* to game was due to the absence of independent verification of ED LOS data. Gaming increased significantly over time (2009-2012) as the *means* to game became more available, usually through the addition or expansion of short-stay facilities attached to EDs. Gaming varied between sites, but those with the highest levels of gaming differed substantially in terms of organisational dynamics and motives. In each case, however, high levels of gaming could be attributed to the strategies of senior management more than to the individual motivations of frontline staff.

**Conclusion:** Gaming of New Zealand’s ED target increased after the real benefits (in terms of process improvement) of the target were achieved. Gaming of ED targets could be minimised by eliminating opportunities to game through independent verification, or by monitoring and limiting the means and motivations to game.

## Background

Gaming has long been identified as a potentially dysfunctional consequence of performance measurement and management systems in the health sector and more generally.^1-3^ There is much discussion about why gaming occurs, and what can be done to minimise and/or prevent it. However, few researchers have explicitly addressed the question of how and why gaming varies between organisations subject to the same performance regime. Asking such a question is important because it can help researchers and practitioners understand the dynamics of gaming within a larger range of implementation contexts, and can assist in developing strategies aimed at minimising gaming, and making judgements about the trade-offs between the benefits and problems associated with performance measurement and management regimes.

In this article, we develop a mixed-methods research approach to the analysis of 4 health organisation case sites over a 6-year period to determine the extent of gaming a mandatory national time target for the completion of emergency department (ED) care in New Zealand, and explain how gaming varied over time and place.

###  Gaming and Variation

#### What Is Gaming?

According to Kelman and Friedman, gaming is ‘making performance on the measured performance dimension appear better when it is not.’^4^ This definition is restricted to identifying behaviour. Mannion and Braithwaite fold the motivation for such behaviour into their definition of ‘altering of behaviour or reporting in order to obtain strategic advantage.’^5^ Zoe Radnor emphasises consequences in defining gaming as the ‘creation of both formal and informal activities which allow a target or regulated deliverable to be met when the result leads to unintended consequences on the external or internal service delivery.’^6^

Definitions of gaming are difficult to clearly separate from broader discussions of cheating, or from other potentially adverse consequences of performance measurement and management, characterised as ‘hitting the target but missing the point.’ Many authors suggest a continuum between gaming and cheating – suggesting that ‘falsification’ is an example of cheating, whereas ‘creative classification’ is gaming.^3,6^ Hitting the target but missing the point covers a much wider range of phenomena, including ratchet effects, effort substitution and myopia.^3^ These can be considered as possible emergent behaviours that arise from regimes of performance measurement. For our purposes, we situate gaming within this broader category of ‘hitting the target but missing the point,’ and regard cheating (data falsification) as one possible manifestation of gaming.

Clearly the fact that some definitions identify problematic consequences points to widespread concerns about gaming behaviour, including ‘breach of trust,’^5^ undermining of the integrity of performance management systems,^4^ and even possibly concealing practices that are hazardous and endanger patients and staff.^7^

### The New Zealand Emergency Department Target

The issue of ED crowding became a prominent health policy issue in the first decade of the 20-first century in many countries.^8^ In 2009, the New Zealand government initiated a Shorter Stays in Emergency Department (SSED) target which stipulated that 95% of patients would be admitted, discharged or transferred from an ED within 6 hours in all District Health Boards (DHBs).^9^ DHBs are the publicly-funded provider organisations responsible for hospital services. The SSED target was part of a wider regime of 6 health targets.^10^ From 2009 to 2017, each of the 20 DHBs reported on their target performance every three months, and the Ministry of Health publicised their performance through local newspapers and its website. After a change of government in 2017, public reporting of the target ceased although it was still a requirement for DHBs to monitor their performance against the measure.

There were many resemblances to the English regime of targets implemented in the early 2000s. The English accident and emergency (A&E) target was part of a more elaborate system of reward and punishment using a combination of reputational ratings and financial incentives and penalties.^11-13^ Hospitals were strongly motivated by these sticks and carrots.^14^ In the English ED target senior managers suffered reputational and financial consequences for poor target performance.^3^ By contrast, in New Zealand top down pressure was exerted informally by the Minister of Health to DHB chief executives when performance against the target was deemed to be unsatisfactory.^10^ Nevertheless, the English experience is instructive for analysing the implementation of the New Zealand target, particularly as it helps identify the range of possible gaming behaviours that are associated with such a target.

### Why Might Gaming the ED Target Vary Between Organisations and Over Time

In exploring reasons for gaming, we start from the contention that context matters in the implementation of performance management regimes.^15-17^ In publicly funded health systems with many local organisational sites or units, we can expect specific instruments of performance management to be applied in differing ways, with differing consequences across different places or times. However, academic research focusing on the nature of contextual variation in gaming is rare.^18^

#### Motives, Opportunities and Means

Gaming is difficult to research because it is usually hidden.^19^ Consequently, some researchers deploy terminology likening gaming to criminal or illegal activity – particularly the language of ‘motive and opportunity.’^1,3^ These are features that may vary significantly between implementation sites as different organisations make sense of the target in different ways.

##### Motives

One possible source of variation is that the problem or condition that is subject to performance management may be objectively more problematic for some organisations than others. Therefore, the motivation to game may be stronger where the problem is more serious. Consequently, organisations starting with low levels of performance may be more likely to engage in gaming.^14^

One strand of literature on gaming focuses on moral attributes of managers and frontline staff. Gwyn Bevan, for example, adopted Julian Le Grand’s language of ‘knights’ and ‘knaves,’^20,21^ which suggests that knights – motivated by more intrinsic factors – may be less inclined to manipulate data than knaves who are more responsive to extrinsic incentives.^22^ Variation in gaming could possibly be explained by different distributions of knightly and knavish behaviours across different implementation sites.

Other authors focus more on organisational dynamics and the relationships between frontline staff, senior management and external agencies. When the weight of implementation is directed downwards to the clinical and clerical staff, street-level bureaucrats may decide to game as part of an overall coping strategy.^23,24^ Organisational approaches that involve ‘pushing the pressure down’ may stimulate gaming because frontline staff may have few other options to meet performance measurement goals.^19^ Frontline staff may fabricate data because they do not believe the regime is legitimate (and experience distance from senior management), and/or have different values than their managers.^25^ Similarly, gaming may also be a product of political pressure, particularly when the local implementers did not see the policy as being aligned with the problem it was trying to address.^26^ Research on English A&E target implementation suggested that hospitals where frontline clinicians bore the weight of implementation were more prone to gaming, in contrast to those adopting a whole-of-hospital approach.^27^

Gaming behaviours can also be directly initiated, organised and designed by senior management.^28^ In such cases, frontline gaming behaviour reflects strategies initiated at senior levels, rather than being simply a reactive type of behaviour. These strategic approaches by senior management may reflect their own judgements of the legitimacy of the performance management regime, and are likely to be shaped by internal organisational factors such as culture and history.^29^

##### Opportunities

Opportunities are the second component of the criminal analogy. Some organisations may have greater opportunities to fabricate data, or engage in creative classification than others. This may be due to varying exposure to independent audit, or to whether or not organisations adopt internal systems that are capable of detecting gaming and cheating.^1^

A classic example of exploiting gaming opportunities was practice of ‘ambulance ramping’ (keeping arriving patients in ambulances to delay the clock starting) that was common in England.^11^ Some data falsification at A&E entry and exit points was also revealed by independent audits of length of stay.^30-32^ Control over the ‘exit’ time meant that staff could record that a patient had been seen, treated, admitted or discharged, when they were still in the A&E awaiting some activity. This behaviour is referred to as ‘clock-stopping.’

##### Means

Building on the criminal analogy, other performance management researchers have made the distinction between ‘means’ and ‘opportunities’^18^ – the analogy being that one might have the opportunity to commit a murder, but not have access to a murder weapon. In the context of ED target implementation, *means* refers to places where patients at risk of breaching the target can be ‘decanted.’ As the majority of patients that stay longer in EDs are admitted to inpatient wards, these wards are one obvious destination for decanting. However, the lack of available beds in these wards is often the primary cause of ED crowding, so this option may not be available for organisations. Another ‘decant’ option is that of the short-stay unit (SSU) where patients are not (yet) admitted, but are held for further observation. Locker and Mason^30^ identified SSUs as a key way in which English hospitals managed their target. SSUs have become standard features of New Zealand hospitals since the mid-2000s.

## Research Question(s) and Methods

The central research questions addressed in this paper are:

1. Was New Zealand’s ED target gamed?
2. To what extent did gaming the New Zealand’s ED target vary between organisations and over time?
3. How can this variation be explained in terms of motives, opportunities and means to game?

To answer these questions, we adopted a mixed-methods approach built on comparative case studies. A mixed-methods approach is most appropriate when the policy impact of interest can be meaningfully measured.^33^ This mixed-methods approach to researching gaming can avoid some pitfalls inherent in approaches that only adopt qualitative or quantitative approaches.

We chose a case study approach as the most appropriate way to underpin a mixed-methods approach.^34^ This comparative approach has also been productively applied in health services research.^35,36^ Our guiding approach to data collection, analysis and interpretation was to use the ED lengths of stay (ED LOS) data to build a picture of *what* and *how much* gaming-related behaviour occurred over time at each case site (Questions 1 and 2), and then apply a secondary analysis of a qualitative (interview) dataset to help explain *why* the extent of gaming differed across time and place (Question 3). These questions required different types of data that, taken together, could present a more comprehensive understanding of gaming and its variation.^37^

###  Case Study Selection

At the outset of our research in 2010, four case study hospital sites chosen were predominantly larger, urban and regional hospitals where problems of ED crowding were more likely to manifest.^34^ We also chose cases that varied in terms of the ED LOS target measure prior to the beginning of policy implementation in mid-2009. The 4 case study hospitals accounted for over a quarter of total ED presentations in New Zealand over the 2006-2012 period.^38^ We also gathered contextual data on the cost of implementing the target using a survey administered to all hospitals in New Zealand in 2011 and 2012.^34,39^ Key features of contextual variation between implementation sites are summarised in Table 1.

**Table 1:** Shorter Stays in ED Case Study Hospital Site Characteristics

	**District Population Size (2013)**		**Performance on Target Measure Prior to Implementation (April-June 2009) (Ministry of Health Figures)**
Hospital 1	100-200 000	Small urban centre	80.7%
Hospital 2	>400 000	Large urban centre	78.7%
Hospital 3	200-400 000	Medium-sized urban centre serving regional population	62.6%
Hospital 4	>400 000	Large urban centre	55.5%

Abbreviation: ED, emergency department.

### Emergency Department Length of Stay Data

Our answers to the first two research questions are based on extensive data on ED LOS was collected over a 6-year period (2007-2012) so that the period of target implementation (2009-2012) could be understood in the context of, and compared with pre-target figures.^34^ The quantitative analysis was conducted between 2014 and 2016 after a thorough process of data cleaning to ensure consistency across sites.

From the data on reported ED LOS distributions, there are some established indicators that constitute evidence of gaming, and allow us to track variation between case sites and over time.^30-32^ We used three indicators of gaming to compare the 4 sites.

Firstly, we measured ‘terminal digit preference bias’ (TDPB). For this indicator we analysed the subset of patients with a recorded ED LOS between 360 and 369 minutes, and measured the percentage of these patients in which the last digit was ‘0’ (ie, had a recorded length of stay of 360 minutes). We would expect this figure would be very close to 10%, as would also be expected for all other terminal digits (1-9). Gaming is indicated when the percentage of terminal digits recorded as ‘0’ significantly exceeds 10% for the increment that includes the target. The higher the percentage of ‘0’s, the higher the level of gaming.

Our second measure is the extent of ‘spikes’ in the distribution of ED wait times around the target of LOS. Locker and Mason identified this phenomenon in English LOS data in the subcategory of patients that were admitted to hospital.^40^

Locker and Mason showed spikes graphically, but we have developed a way of measuring them. To develop this indicator, LOS data for the subcategory of ED patients that were admitted to inpatient wards was divided into equal time increments of 15 minutes, and the proportion of patients with recorded waiting times within each increment is plotted against the y-axis as a percentage of all presentations. The height of the spike equals *a-[( b+c )/2* ], where *a* is the percentage of all admitted patients with an ED LOS between 346 and 360 minutes (the increment that includes the ED target), *b* is the percentage of admitted patients with a 331-345 minute LOS (the pre-target increment), and *c* is the percentage for 361 to 375 minutes (the post-target increment). For example, if the target increment is 9%, the pre-target increment is 4%, and the post target increment is 2%, the spike height is 6.

This measure is a useful, but more indirect indicator of gaming. First, spikes indicate the size of the potential pool for the inaccurate recording of the end of the ED visit that we see in TDPB figures. Second, the height of the spike can also indicate the pool of potential opportunities to decant patients to other destinations (particularly SSUs) in order to meet the target, rather than for clear clinical reasons.

Our third indicator of gaming is the percentage of patients recorded as having a very short (0-15 minute) length of stay in ED. This indicator emerged from our analysis of the ED LOS patterns. This spike indicates the extent to which patients referred to ED by their general practitioner were immediately transferred to an SSU. While this is a legitimate clinical practice, we classify it as gaming in the context of the ED target because these patients should not be counted as ED presentations, as they are not cared for by ED staff, and do not occupy space in the ED.

### Case Study Interview Data

We then turned to a source of qualitative data in order to help explain the patterns identified in the ED LOS data. One author (LC) had conducted 68 face-to-face semi-structured interviews about the implementation of the ED target as part of PhD research into processes of implementation of the ED target. This researcher had a background in ED nursing and health service management. Interviewees were nursing, medical and allied health clinicians, and clinical and non-clinical managers in the hospital and DHB organisations (see Table 2). Participants were recruited by posting invitations on DHB intranets, and by identifying prospective interviewees with the assistance of key contacts at each site. Interview data were collected in two rounds in early 2011 (47 interviews) and mid-2012 (21 interviews), with thirteen participants interviewed in both rounds.

**Table 2:** Distribution and Characteristics of ED Target Implementation Case Study Interviews

	**ED Clinicians**	**ED Clinician-Managers**	**ED Managers**	**Inpatient Clinicians**	**Inpatient Clinician-Managers**	**Hospital/ Organisational Managers**	**Total**
Hospital 1	5	1	2	6	1	1	16
Hospital 2	2	1	2	4	2	7	18
Hospital 3	6	1	1	5	1	3	17
Hospital 4	3	2	3	5	1	3	17
**Total**	**16**	**5**	**8**	**20**	**5**	**14**	**68**
Round 1 (2011)	11	4	4	13	5	10	47
Round 2 (2012)	5	1	4	7	0	4	21

Abbreviation: ED, emergency department.

The interviewer did not ask specific questions about gaming during the interviews. This data was collected before we developed our analysis of ED length-of-stay gaming indicators. Nevertheless, the interviews were directly relevant, particularly as they provided a rich source of information about the different organisational contexts and conditions in which implementation of the ED target transpired. In order to find relevant information we analysed a summary of the interview findings prepared by the interviewer in 2014, and in 2017 we conducted text searches of the interview transcripts for particular terms and phrases that could refer to gaming. These terms included ‘clock-stopping,’ ‘gaming,’ and ‘fudging.’ As such, all information about gaming was volunteered in the context of the wider interview.

## Results

### Overview of Change in ED LOS Data Over Time

Table 3 provides some background to our findings on gaming by summarising key features of ED target implementation in the case study sites reported in previous research.

**Table 3:** Changes in Target Performance in Case Study Sites

	**Performance on Target Measure Prior to Implementation (April-June 2009) (Ministry of Health Figures)**	**Performance on Target Measure for October – December 2012)** ^38^	**Growth in ED Presentations** ^38^ **2009-2012**	**Estimated Cost of ED Target Implementation Per ED Presentation (2009-2012)** ^39^
Hospital 1	80.7%	94.9%	Low (<10%)	$6.44
Hospital 2	78.7%	96.2%	High (>15%)	$15.14
Hospital 3	62.6%	84.8%	High (>15%)	$17.42
Hospital 4	55.5%	97.8%	High (>15%)	$32.32

Abbreviation: ED, emergency department.

### Evidence of Gaming From ED LOS Data

**Figure 1:**
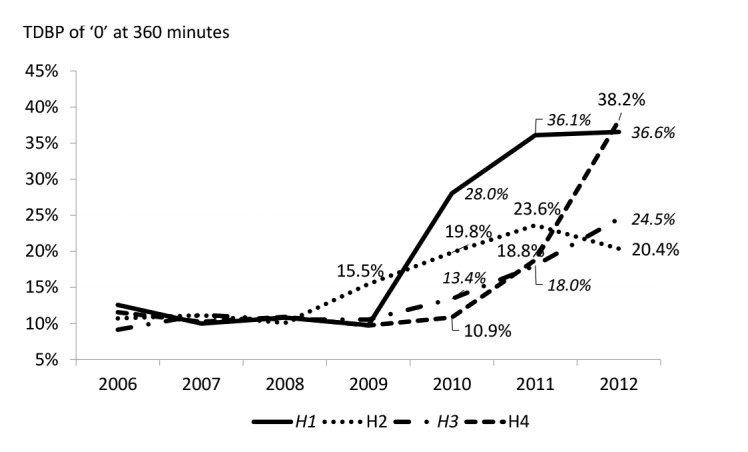
TDPB of ‘0’ at 360 Minutes in Case Study Hospitals 2009-2012. Abbreviation: TDPB, terminal digit preference bias.

Figure 1 shows that TDPB appeared after the 2009 introduction of the ED target in all case study sites with the exception of H2 which initiated its own 6-hour target in 2008.

H1 shows the largest and steepest increase in TDPB after the introduction of the target in 2009. H2 demonstrates a much ‘flatter’ increase, albeit one that began earlier, peaking at 24% in 2011. H3 and H4 both show small increases in TDPB before 2011, after which the rate rises more steeply, particularly for H4.

**Figure 2:**
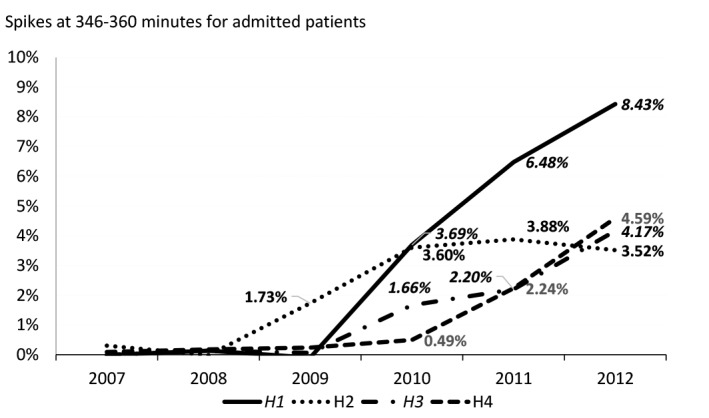
Height of Spikes for 346-360 Minutes in Case Study Hospitals 2009-2012 (Admitted Patients).

Figure 2 shows the 346-360 minute spikes from 2009 to 2012 for the case study hospitals. In 2009 there are no discernible spikes in any of the hospitals apart from a small one in H2. By 2012 all sites had demonstrated spikes for the 346-360 minute interval – H2, H3, and H4 each had spikes of around 4% and the figure for H1 was over 8%.

The height of the spikes increases over the 2009-2012 period in each hospital, apart from a slight decrease for H2 in 2012. Both H3 and H4 show slowly increasing spikes from 2009 to 2011, followed by a much steeper increase from 2011 to 2012, particularly for H4.

**Figure 3:**
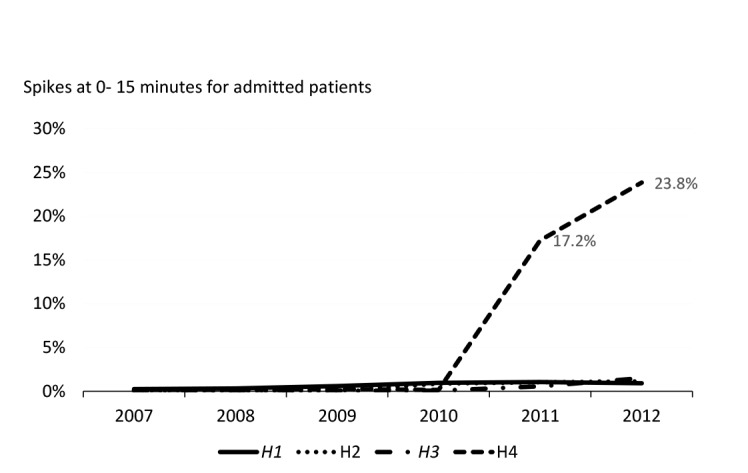
Percentage of Admitted Patients With 0-15 Minutes ED LOS. Abbreviation: ED LOS, ED lengths of stay.

This practice was only observable in H4. In 2012, 24% of all ED patients in H4 had a recorded ED LOS of less than 15 minutes (see Figure 3). This practice made it significantly easier for H4 to meet the 95% target by inflating the overall number (the denominator) of patients recorded as passing through ED. The data presented in this figure include a small amount of patients that were redirected to alternative acute care providers prior to assessment, but still counted for target purposes.

Our answers to the first 2 research questions on the extent and variation in gaming over time and place are summarised in Table 4. We have divided the table into 2 parts corresponding to early and late implementation. In the early phase of implementation, gaming behaviours were most extensive in H1 and H2, but by the later period (2011-2012), H4 joined H1 as the most prominent gaming site.

**Table 4:** Variation in Extent of Gaming Across Time and Case Study Sites

**Early implementation (2009-2010)**				
	**Hospital 1**	**Hospital 2**	**Hospital 3**	**Hospital 4**
Official target performance	Relatively high	Relatively high	Low	Low
Gaming Indicators
TDPB ‘0’ at 360-369	High	Moderate	Low	Low
Spike 346-360	Moderate	Moderate	Low	Low
Spike 0-15	None	None	None	None
**Late implementation (2011-2012)**				
Official target performance	Achieved	Achieved	Not achieved	Achieved
Gaming Indicators
TDPB ‘0’ at 360-369	Very High	Moderate	Moderate	Very High
Spike 346-360	Very High	Moderate	Moderate	Moderate
Spike 0-15	None	None	None	Very High

Abbreviation: TDPB, terminal digit preference bias.

### Case Study Interview Data

We now turn to the interview data collected at the time of target implementation to help explain why gaming increased over time in all sites, and why it was highest in H1 and H4. This is a puzzle because these sites were opposite in every respect in terms of the contextual data (population size, growth in ED presentations, amount spent on target implementation, official target performance) reported in Tables 1 and 3.

#### Hospital 1

In this case site, pressure to meet the target was intense and focused tightly on the ED rather than the wider hospital. From 2009, a new Chief Executive of the DHB responsible for H1 was strongly focused on the ED target. Early strategies focused on using existing space in the ED, and creating new spaces to decant patients on their way to inpatient wards. The CEO exerted considerable direct pressure on ED medical and nursing staff.^41^

Interviewees at this site described a range of gaming behaviours. One staff member described ‘clock-stopping’ behaviour - the falsifying the record of time of departure from ED as follows:

*“…the decision to discharge or transfer the patient had been made but the patient was taken off the acute [ED target] stream before they were moved from the unit”* (Hospital 1, Round 1, Participant C).

Respondents also described two strategies which involved decanting patients to parts of the hospital outside the target stream. One of these was within the ED itself.

*“We did create 4 ED observation beds in the assessment unit…they’re managed by the emergency team. And patients who you want to keep an eye on them overnight; and they don’t need to be admitted medically, they’re put into our ED observation – they’re admitted patients, um, but they’re managed by the emergency team. As soon as they are admitted into the system as an ED obs patient, then they come off the target”* (H1, R1, Participant D).

This created the opportunity for gaming as described by another respondent:

*“I also cover the acute assessment unit so we find that sometimes the AAU [acute assessment unit] is a dumping ground for people because the 6 hours target’s coming; we’ve got to put them somewhere so we’ll just quickly move them to another area. So we think that that’s not quite meeting the target, but it’s just moving them out of one area to another”* (H1, R1, Participant A).

From 2011 onwards, ED staff in H1 began to move surgical patients to the acute assessment unit within the ED in order to stop the clock. For example, if a patient came in at 10 pm they would stay in the ED until nearly 4 am, then they would be transferred to the AAU because there were no surgical clinical registrars working overnight.

#### Hospital 2

H2 was one of the first hospitals to respond to the target. This site quickly developed a ‘whole of hospital’ strategy to reduce ED waiting times from 2008 (when the organisation instituted its own 6-hour target). This site established a whole-of-hospital target governance group as well as an operational group consisting of senior clinical leaders across the hospital. Specific initiatives undertaken included the introduction of a discharge ward in the surgical unit, a new hospital operations management unit, and ‘cohorting’ of patient groups in wards designed to improve staff and bed utilisation.

This whole of hospital strategy was recognised by many respondents, including those on the frontline:

*“I can’t help but think part of the success is, has been, the organisational involvement, you know, because we’ve struggled with this for so many years from ED, real, just ground level, and I think the real success is having an organisation that really embraces it a bit”* (H2, R1, Participant G).

Interviews with staff at H2 also provided clear evidence of clock-stopping, described here as ‘falling off the screen’ and ‘black holes.’

*“[The nurses] they’ll say to me is that patient not up yet [on the ward]? And they go “we’ve rung them [ED] they haven’t come back to us” so I don’t know where the patient is or what. Then it’s the whole information screen thing where they fall off the screen and I go if they’ve fallen off the screen they must be on their way, and there’s that black hole from when the patient sort of leaves ED before they get here”* (H2, R1, Participant I).

This behaviour was attributed to pressure from hospital management. According to one respondent, frontline staff engaged in gaming, even though they were uncomfortable with it.

*“If a patient is coming close to their 6 hour mark the, you know the Charge Nurses come out, the managers come out, the doctors get a rark up, a lot of the time we’re just waiting on patient to go up to the ward, so the patient will be scanned out of the department before they reach the 6 hour mark even if they haven’t actually physically left the department, which, I actually find quite unsafe and I don’t like that practice but, that’s what the Charge Nurses do”* (H2, R1, Participant L).

#### Hospital 3

This hospital struggled to progress towards the ED target throughout the 2009-2012 period. Participants thought that the target was primarily framed as an ED responsibility rather than focus of the whole of hospital or whole of organisation. According to one ED clinician:

*“Because it was called an ED target, I think it’s kind of branded as we own it, and so that has, that has resulted in some of that resistance from the rest of the organisation...I’ve sat here with the clinical director from paediatrics and he said to me it’s your target, you guys made the decision that 6 hours was the timeframe”* (H3. R1, Participant C).

As with the other sites, clock-stopping behaviour was identified.

*“ So there was things like – right take that patient off the board – they’re about to go to the ward and I’m just waiting for the attendant, but take them off, you know. And 20 minutes later it’s like this patient’s still here. Oh well the attendant hasn’t arrived”* (H3, R1, Participant K).

Interviewees in H3 regarded the opening of new ED and short-stay facilities in 2011 as the best opportunity to improve target performance. From the second round of interviews, there was a continued struggle to gain buy-in and momentum on the target contributed to by the multiplicity and complexity of medical specialty service and professional groups, as well as persistent issues regarding hospital leadership. As such, implementation still defaulted to the ED.

Interviewees described moving patients from the ED to a SSU for medical patients in order to avoid target breaches. According to a Senior Medical Officer, pressure to game increased after 2011:

*“… there’s possibly one thing that’s changed since the last time we spoke is the little bit more pressure to game, but only on a subtle thing, mainly on use of short stay and observed spaces…. I mean that’s just a manifestation of the pressure that we’re under to achieve it”* (H3, R2, Participant H).

However there was some clinician resistance to this pressure to game. One participant, who described the clock-stopping behaviour in relating to patients admitted to wards, went on to say.

*“You know, things like that; so there was a little bit of fudging which I didn’t agree with and I had great difficulty with and that’s probably when I started to say things like stuff the target, you know – we need to know who is here, so don’t take them off”* (H3, R1, Participant K).

#### Hospital 4

In H4, a new, expanded ED facility, with associated SSU capacity, opened in early 2011. Performance against the target remained low until this point in time. In common with all other sites, clock-stopping was identified in H4.

*“[Interviewer: What do you mean by fudging?] Well they’ll move patients before they’ve actually gone and that will stop the clock, you see. Or, they’ll take people off the computer before they’ve been discharged because they’re intended to go but they’re waiting for an ambulance but they’re still under our care”* (H4, R1, Participant B).

Respondents in round 1 also commented on the lack of leadership and engagement from senior managers, and a lack of support for frontline implementation of the target. However, from 2011 target implementation became a priority across the whole organisation, and senior leadership and support was clearly visible at the frontline. One respondent described this change:

*“And the other thing I think that’s really made a big contribution is better engagement from the organisation. The organisation at the top…we hadn’t moved [into the new department], so even at that point and the 6 months prior to that there wasn’t the engagement at the top”* (H4, R2 Participant D).

In the second round of interviews, it was clear that the availability of the SSU (referred to as the Alpha ward) was playing a significant part in target implementation:

*“Respondent: Oh, well of course that [the Alpha ward] that’s helped immensely because it’s somewhere that you can send your patients to, so um the patients that need med, surg , ortho gynae input so it might be a patient that needs to go to a ward, but there isn’t a ward bed available and then by way of preventing a breach, you can move them into the Alpha ward”* (H4, R2, Participant D).

Table 5 provides a synthesis of the qualitative data in terms of the motivation, opportunities and means.

**Table 5:** Variation in Motivation, Opportunities and Means of Gaming Across Time and Sites

**Early implementation (2009-2010)**				
	**Hospital 1**	**Hospital 2**	**Hospital 3**	**Hospital 4**
Motivation	High pressure on ED from senior leadership	Whole of organisation approach to ED target, management pressure on clinical staff	Target is ED’s problem Intermittent pressure from leadership Some clinical resistance to pressure to game	Lack of senior leadership on target implementation, little pressure on ED
Opportunities	All sites report ‘clock-stopping’ and ‘patients disappearing from screen’
Means	Decant to Acute Assessment Unit and/or ward corridors	Decant to ED SSU and/or wards	Decant to ED SSU (medical only) and/or wards	Decant to ED SSU and/or wards
**Late implementation (2011-2012)**				
Motivation	High pressure on ED staff from senior leadership which views target as an important priority	Whole of organisation approach to ED target, management pressure on clinical staff, moderate alignment of target with organisational perception of problem	Target is ED’s problem Intermittent pressure from senior leadership, clinical resistance to gaming pressure, low alignment with organisational challenges	Whole of organisation approach to ED target, moderate alignment of target with organisational perception of problem
Opportunities	All sites report ‘clock-stopping’ and ‘patients disappearing from screen’
Means	Decant to Acute Assessment Unit and/or ward corridors	Decant to ED SSU and/or wards	Decant to ED SSU (medical only) and/or wards	Decant to ED SSU and/or wards GP referrals straight to SSU

Abbreviations: ED, emergency department; SSU, short-stay unit.

## Discussion

Taken together, the data on gaming indicators and interview extracts demonstrate that clinicians and managers in all sites developed gaming behaviours and strategies in response to the ED target. Here we bring together these data in order to understand how variation can be explained in terms of motivations, opportunities and means (research question 3).

### Opportunities to Game Were the Same Across All Sites

Our qualitative data conclusively confirmed that gaming behaviour was clearly detected in all sites. Our interviews provided accounts of how it happened. Respondents from all sites described clock-stopping behaviour in which patients were recorded as having left the ED even though they remained in the ED. Operational staff recorded both the time of arrival and the time of departure from ED for each patient, providing substantial opportunity for data manipulation. A further opportunity for data manipulation occurred after data collection and prior to DHBs submitting figures to the Ministry of Health. There was no system of independent audit of ED length-of-stay data, ensuring that any fabrication of data would not be discovered.

###  The Means to Game Developed Over Time

All sites also reported the practice of transferring patients to SSUs (or the AAU in the case of H1) or inpatient wards in order to avoid target breaches. Our ED LOS data shows that this practice increased over time.

During the implementation period, 3 of the 4 sites (H2, H3, and H4) acquired increased capacity to decant patients to an SSU. Increases in gaming in H3 and H4 occurred *after* the opening of expanded EDs and associated additional capacity in SSUs in early 2011. The increase was more marked in H4 because its SSU covered all patients, whereas it was only used for medical patients in H3. The same increase occurred earlier in H2.

In contrast, H1 engaged in significant gaming even though the availability and capacity of destinations for decanting patients was limited to a small number of beds in an ED acute assessment unit, and corridors in inpatient wards. The differences in capacity to decant patients matches the differences in resources allocated to ED target implementation in the last column of Table 1. Therefore it is plausible to suggest that H1 relied more heavily on gaming behaviours such as moving patients to ward corridors because it had *less* capacity to increase levels of staff and beds. In all other sites, higher levels of gaming were associated with *greater* available capacity to decant patients.

### Motivation to Game Varied Across the Sites

The sites with the highest levels of gaming, H1 and H4, were very different, even opposite, to each other in terms of organisational dynamics and motives. High levels of gaming were highly dependent on contextual factors. In H1 it was the downward pressure on ED staff from senior management which caused frontline staff to resort to the only means at their disposal – placing patients in ward corridors, decanting to inpatient wards and the AAU and/or taking ED patients ‘off the screen.’

Conversely, high levels of gaming in H4 emerged as an artefact of senior management’s ‘whole-of-hospital’ strategies to meet the target. These included expanding the scope for very short ED LOS, and utilising availability of a new SSU to manage patient flow between the ED and the inpatient wards. The decision to classify general practitioner-referred patients as part of the target denominator (the 0-15 minute spike) was a strategic rather than an operational decision.

H3 also offers an interesting contrast to H1. H3 was also was characterised by an ED-focused approach to implementation, but gaming behaviour was at lower levels. This was the site where official target performance was comparatively poor. Compared to other sites, senior management did not place as much pressure on ED staff to meet the target, and ED staff appeared less willing to falsify the data.

Another clear finding is that gaming was more prevalent the closer organisations were to reaching the target. Contrary to our expectations from the literature,^42^ poor performance in the early period in H3 and H4 was associated with low levels of gaming, whereas the better performing sites (H1 and H2) gamed more. Gaming increased in H3 and H4 only once new facilities had opened in 2011.

Overall, while the motivations to game were highly context specific across the sites, the strategic behaviour of senior management was a common factor, even though the details of this strategic behaviour varied substantially. Gaming was induced or initiated by the actions of senior managers. As such, accounts of motivation that emphasise the moral attributes of frontline clinicians are not a major factor driving the variation in gaming behaviour. The only location in which knightly or knavish behaviour might have affected rates of gaming was in H3 in which clinicians resisted the (weaker) management pressure at that site.

## Conclusion

Our aim in this paper is to open up the questions of how and why gaming varies across time and place in order to inform discussions about how to minimise gaming of ED targets, and performance management regimes in health more generally. Our findings are themselves bound by time and place, as they are predominantly from larger, urban hospitals. We also acknowledge that the data is over seven years old, and that some important features regarding ED LOS data capture, gaming, and the evolution of service delivery in hospitals, may have changed the ED target landscape after 2012.

However, our key aim was to provide more fine-grained analysis of the ways in which gaming behaviour emerges and evolves in specific organisational contexts in the first years of implementation. This is an approach can be readily applied to researching the implementation of any performance management regime. Gaming behaviours are not static, and are subject to local, contextual influences.

With this in mind, we offer three starting points for minimising the gaming of performance management.

1) The most straightforward way to prevent gaming of targets is to ensure that there are no opportunities to game.^1^ In the case of the SSED target, we are confident that gaming was an inevitable consequence of policy design in which there was no independent system of monitoring and verification. If policy-makers choose not to eliminate opportunities to game, they need to focus on mitigating means and motivation.
2) The SSED story is consistent with literature on the life cycle of performance measures, particularly the argument that performance measures ‘wear out’ over time.^19,43^ If organisations acquire the means to game, then one response is to develop systems to detect and monitor the emergence of gaming.^19^ Monitoring and providing feedback on spikes and TDPB is a practical and easily implemented response. If targets show signs of wearing out after 2-3 years, then some adaptation of measures is advisable.
3) In order to address and minimise motivation to game, custodians of performance management systems should pay attention to the strategies of senior management. These strategic responses differed markedly between the organisations that gamed the most. These responses mattered more than the motivations of frontline clinicians. The challenge is to understand the interplay between the problem perceptions and priorities of external actors and organisations (in this case the Minister and Ministry), senior organisational managers, and front-line staff,^25,26,28^ and how these map on to local patterns of service demand, configuration of services and resourcing decisions.

There is a broader question of whether or not gaming is a necessary evil to be tolerated to some degree in the pursuit of improved health service outcomes and processes. Other research into New Zealand’s ED target demonstrated that there were clear benefits for patients attributable to the target’s introduction^44^ which were likely to be because of improved patient flow.^38^ It is unlikely that these benefits would have been achieved without the ED target.

However, these improvements were manifest in the first 18 months of implementation, *before* gaming behaviour intensified.^38^ As time went on, the adverse consequences of gaming such as loss of staff morale, weakening of intrinsic motivation, and declining trust in the veracity of performance data, became increasingly problematic.^10^ As such, the balance between benefits and dysfunctional effects of the ED target changed over time. Arguably, it is easier for organisations to game than to change entrenched culture attitudes and behaviour. It is unreasonable to expect that an ED LOS target, by itself, can induce the changes of organisational culture and hospital practice that are necessary to address systemic access block. But if we understand how and why gaming intensifies at certain times or in particular places, it may be possible to detect it earlier and develop responses that reap the benefits of an ED target while minimising dysfunctional consequences of gaming.

## Acknowledgements

The Shorter Stays in Emergency Departments National Research Project was a collaboration of a team of researchers from the School of Population Health (Faculty of Medical and Health Sciences, University of Auckland, Auckland, New Zealand) and the Emergency Department Research Team at the Auckland District Health Board. This project was funded by a competitive 3-year grant of NZ$1 138 171.00 from the Health Research Council of New Zealand (HRC 10-588) which commenced on October 1, 2010. The current case study research formed part of that Project. The funding body has had no role in the design of the study, data collection, analysis or interpretation, or writing of this manuscript. The authors would like to acknowledge the contributions to the project of Lisa Walton (Research Fellow), Jayshree Ramesh-Sukha (Project Administrator) and Denish Kumar (Data Manager) and contributions the case study research of Project investigators and advisors not listed here as authors. Our gratitude also goes to the hospital managers, clinicians and staff who took part in case study interviews and survey described in this paper.

## Ethical issues

Ethical approval for this study was granted by the New Zealand Multi-Region Ethics Committee (MEC 10/06/60) on July 1, 2010. Written informed consent as obtained from all survey and interview participants and all survey and interview data was anonymised and encrypted. All data reporting hospital target performance and ED LOS was aggregated data with all identifiers removed.

## Competing interests

Authors declare that they have no competing interests.

## Authors’ contributions

PJ and LMC were the lead investigators, and TT, SA, and PC were investigators for the Shorter Stays in Emergency Departments National Research Project. TT had overall responsibility for the data integration workstream and he reviewed the literature and drafted the manuscript aided by all other authors. PJ had overall responsibility for quantitative data sources assisted by SA. LMC designed and had overall responsibility for the qualitative workstream and data analysis, assisted by TT and PC. All authors read, reviewed and approved the final manuscript.

## Authors’ affiliations

^1^Faculty of Medical and Health Sciences, The University of Auckland, Auckland, New Zealand. ^2^Auckland District Health Board Faculty of Medical and Health Sciences, University of Auckland, Auckland, New Zealand. ^3^Auckland District Health Board, Auckland, New Zealand. ^4^School of Population Health, Faculty of Medical and Health Sciences, University of Auckland, Auckland, New Zealand.

## Key messages

**Implications for policy makers**

- If the design of emergency department (ED) targets allows opportunities for gaming, such opportunities will be exploited by implementing organisations.
- Organisations become more adept at gaming an ED target the longer that it is in place.
- The strategies of senior managers in organisation may be a far more important factor shaping motivation to game than the moral attributes of individual clinicians and front-line staff.
- Policy-makers need to pay closer attention to detecting and responding to gaming of ED targets over time, if targets are to retain their integrity and effectiveness.

**Implications for the public**

Governments often use performance targets for health sector organisations as a way of holding organisations accountable. However, staff in organisations can ‘game’ targets, making performance appear better when it is not. Our research demonstrated that New Zealand hospitals engaged in gaming the Shorter Stays in emergency departments (EDs) target in the three years after the government introduced it in 2009. Performance targets often lose their capacity to reflect actual performance as health services develop and evolve. When there is no system set up for independent verification of ED waiting time figures, the broader public need to be aware of the potential for gaming, and be prepared to question the performance data, particularly when target achievement is linked to the electoral strategies of governing parties. In this way, citizens can help ensure that governments and health sector organisations minimise practices that ‘hit the target but miss the point.’

## References

[1] Van Dooren W, Bouckaert G, Halligan J. Performance Management in the Public Sector. London: Routledge; 2010.

[2] Smith P (1995). On the unintended consequences of publishing performance data in the public sector. Int J Public Adm.

[3] Bevan G, Hood C (2006). What’s measured is what matters: targets and gaming in the English public health care system. Public Adm.

[4] Kelman S, Friedman JN (2009). Performance improvement and performance dysfunction: an empirical examination of distortionary impacts of the emergency room wait-time target in the English National Health Service. J Public Adm Res Theory.

[5] Mannion R, Braithwaite J (2012). Unintended consequences of performance measurement in healthcare: 20 salutary lessons from the English National Health Service. Intern Med J.

[6] Radnor Z (2008). Muddled, massaging, manoeuvring or manipulated? A typology of organisational gaming. Int J Prod Perform Manag.

[7] Mears A, Webley P (2010). Gaming of performance measurement in health care: parallels with tax compliance. J Health Serv Res Policy.

[8] Working Group for Achieving Quality in Emergency Departments. Recommendations to Improve Quality and the Measurement of Quality in New Zealand Emergency Departments. Wellington: Ministry of Health; 2008.

[9] Ardagh M (2010). How to achieve New Zealand’s shorter stays in emergency departments health target. N Z Med J.

[10] Tenbensel T, Chalmers L, Willing E (2016). Comparing the implementation consequences of the immunisation and emergency department health targets in New Zealand. J Health Organ Manag.

[11] Bevan G, Hood C (2006). Have targets improved performance in the English NHS?. BMJ.

[12] Jones P, Schimanski K (2010). The four hour target to reduce Emergency Department ‘waiting time’: a systematic review of clinical outcomes. Emerg Med Australas.

[13] Boyle A, Mason S (2014). What has the 4-hour access standard achieved?. Br J Hosp Med (Lond).

[14] Bevan G, Hamblin R (2009). Hitting and missing targets by ambulance services for emergency calls: effects of different systems of performance measurement within the UK. J R Stat Soc Ser A Stat Soc.

[15] Pawson R. Evidence-Based Policy: A Realist Perspective. London: Sage; 2006.

[16] Sanderson I (2006). Complexity, ‘practical rationality’ and evidence-based policy making. Policy Polit.

[17] Pollitt C. Context in Public Policy and Management. Cheltenham, Glos, GBR: Edward Elgar Publishing; 2013.

[18] Drew J, Grant B (2017). Means, Motive, and Opportunity – Local Government Data Distortion in a High-Stakes Environment. Aust J Public Adm.

[19] Pollitt C (2013). The logics of performance management. Evaluation.

[20] Le Grand J. Motivation, Agency and Public Policy: Of Knights & Knaves, Pawns & Queens. Oxford: Oxford University Press; 2003.

[21] Bevan G (2010). Performance measurement of “knights” and “knaves”: differences in approaches and impacts in British countries after devolution. Journal of Comparative Policy Analysis:Research and Practice.

[22] Janus K (2014). The effect of professional culture on intrinsic motivation among physicians in an academic medical center. J Healthc Manag.

[23] Lipsky M. Street-Level Bureaucracy: Dilemmas of the Individual in Public Services. Cambridge: MIT Press; 1980.

[24] McCann L, Granter E, Hassard J, Hyde P (2015). “You Can’t Do Both—Something Will Give”: Limitations of the Targets Culture in Managing UK Health Care Workforces. Hum Resour Manage.

[25] Groth Andersson S, Denvall V (2017). Data Recording in Performance Management: Trouble With the Logics. Am J Eval.

[26] Boswell C, Rodrigues E (2016). Policies, politics and organisational problems: multiple streams and the implementation of targets in UK government. Policy Polit.

[27] Weber EJ, Mason S, Carter A, Hew RL (2011). Emptying the corridors of shame: organizational lessons from England’s 4-hour emergency throughput target. Ann Emerg Med.

[28] Lægreid P, Neby S (2016). Gaming, Accountability and Trust: DRGs and Activity-Based Funding in Norway. Financial Accountability & Management.

[29] Mannion R, Harrison S, Jacobs R, Konteh F, Walshe K, Davies HT (2009). From cultural cohesion to rules and competition: the trajectory of senior management culture in English NHS hospitals, 2001-2008. J R Soc Med.

[30] Locker TE, Mason SM (2006). Are these emergency department performance data real?. Emerg Med J.

[31] Locker T, Mason S, Wardrope J, Walters S (2005). Targets and moving goal posts: changes in waiting times in a UK emergency department. Emerg Med J.

[32] Locker TE, Mason SM (2006). Digit preference bias in the recording of emergency department times. Eur J Emerg Med.

[33] Greene JC. Mixed Methods in Social Inquiry (Vol. 9). San Fransisco, CA: Jossey-Bass; 2007.

[34] Jones P, Chalmers L, Wells S (2012). Implementing performance improvement in New Zealand emergency departments: the six hour time target policy national research project protocol. BMC Health Serv Res.

[35] Denis JL, Lamothe L, Langley A (2009). The reciprocal dynamics of organizing and sense-making in the implementation of major public-sector reforms. Can Public Adm.

[36] Ferlie E, Fitzgerald L, McGivern G, Dopson S, Bennett C. Making wicked problems governable?: the case of managed networks in health care. Oxford, United Kingdom: Oxford University Press; 2013.

[37] Greene JC (2015). The emergence of mixing methods in the field of evaluation. Qual Health Res.

[38] Tenbensel T, Chalmers L, Jones P, Appleton-Dyer S, Walton L, Ameratunga S (2017). New Zealand’s emergency department target - did it reduce ED length of stay, and if so, how and when?. BMC Health Serv Res.

[39] Jones P, Sopina E, Ashton T (2014). Resource implications of a national health target: The New Zealand experience of a Shorter Stays in Emergency Departments target. Emerg Med Australas.

[40] Locker TE, Mason SM (2005). Analysis of the distribution of time that patients spend in emergency departments. BMJ.

[41] Chalmers L. Inside the Black Box of Emergency Department Time Target Implementation in New Zealand. Auckland: Health Systems, School of Population Health, University of Auckland; 2014.

[42] Hamblin R (2008). Regulation, measurements and incentives The experience in the US and UK: does context matter?. J R Soc Promot Health.

[43] Van Thiel S, Leeuw FL (2002). The Performance Paradox in the Public Sector. Public Perform Manag Rev.

[44] Jones P, Wells S, Harper A (2017). Impact of a national time target for ED length of stay on patient outcomes. N Z Med J.

